# Effect of Uterine Weight on the Surgical Outcomes of Robot-Assisted Hysterectomy in Benign Indications

**DOI:** 10.7759/cureus.56602

**Published:** 2024-03-20

**Authors:** Naofumi Higuchi, Kiyoshi Kanno, Yoshifumi Ochi, Mari Sawada, Shintaro Sakate, Shiori Yanai, Masaaki Andou

**Affiliations:** 1 Department of Gynecology, Kurashiki Medical Center, Kurashiki, JPN

**Keywords:** benign indications, uterine weight, minimally invasive surgery, hysterectomy, robotic surgery

## Abstract

Background

Uterine weight is an important factor in determining the complexity of a hysterectomy. Although greater uterine weight increases operative time and blood loss in open or laparoscopic surgery, it remains uncertain whether this applies to robot-assisted hysterectomy. This study aimed to investigate the effect of uterine weight on the surgical outcomes of robot-assisted hysterectomy.

Methods

We conducted a retrospective cohort study involving 872 patients who underwent robot-assisted hysterectomies at our institution between January 2019 and June 2022. Of these, 724 cases were analyzed and classified into four groups based on uterine weight: <250 g (377 patients), 250-500 g (253 patients), 500-750 g (69 patients), and ≥750 g (25 patients). We performed univariate analysis with the following endpoints: operation time, blood loss, postoperative hospital stay, complication rate, conversion to laparotomy rate, and blood transfusion rate.

Results

Operating time and blood loss increased significantly with greater uterine weight in the four groups (both p-values <0.01), but postoperative hospital stay and complication rate did not increase (p = 0.448, p = 0.679, respectively). None of the patients underwent conversion to laparotomy or blood transfusion.

Conclusion

Although the operating time for robot-assisted hysterectomy and blood loss increased with greater uterine weight, the complications and length of postoperative hospital stay were similar between groups. Robot-assisted hysterectomy is safe in cases of much uterine weight.

## Introduction

Robot-assisted hysterectomies have been performed worldwide since the emergence of surgical robots. The difficulty of robot-assisted hysterectomy is determined by various factors, such as uterine weight, patient obesity, endometriosis, and adhesions from previous surgeries [1,2]. Uterine weight, in particular, varies from a small uterus to a large uterus weighing more than 1 kg [3,4], depending on the indications. Most previous studies have reported that surgical difficulty increases with increasing uterine weight, suggesting that uterine weight affects surgical outcomes, such as operative time, blood loss, and conversion to laparotomy [5-8]. However, the impact of uterine weight on the surgical outcomes of robot-assisted hysterectomy has not been elucidated, with previous studies showing disparate results.

Further, although many researchers agree that increased uterine weight increases operative time and blood loss [5-8], this is not so laparotomy conversion; some believe the risk of conversion increases with uterine weight above 750 g [5], while others believe conversion is unrelated to uterine weight [7]. Moreover, the specific uterine weight that worsens surgical outcomes remains unknown [9]. Therefore, using data from the largest sample ever recorded at our high-volume center, this study aimed to clarify the effect of uterine weight on the surgical outcomes of robot-assisted hysterectomy.

## Materials and methods

We conducted a retrospective cohort study of patients who underwent robot-assisted hysterectomy at the Kurashiki Medical Center, Kurashiki, Japan, between January 2019 and June 2022. All patients were preoperatively evaluated for lesions using internal examinations, transvaginal ultrasound tomography, and magnetic resonance imaging (MRI). There were seven console surgeons with da Vinci certification during the study period, all of whom had lots of experience in laparoscopic surgery and then applied the experience to robotic surgery. In this study, we used data from robot-assisted hysterectomy performed by these seven surgeons. Exclusion criteria included malignant disease, severe endometriosis (revised American Society for Reproductive Medicine [rASRM] classification stage III or IV [10]), and simultaneous surgery such as sacrocolpopexy, extensive adhesiolysis for previous surgery, and removal of deep endometriosis lesions. Because we planned for the study to be a univariate analysis, we excluded patients with severe endometriosis because severe endometriosis is considered a confounding factor for uterine weight [1,2]. However, endometriosis without extrauterine extension (uterine adenomyosis alone) was included. In the case of hysterectomy with adnexectomy, the weight of the uterus was measured, excluding the weight of the adnexa. Data were collected after obtaining informed consent from all patients, and approval was obtained from our institutional review board (no. 1424).

We describe robot-assisted hysterectomy performed at our institution using the da Vinci Xi (Intuitive Surgical Inc., Sunnyvale, CA, USA). The docking of the robot was performed from the right side of the patient. Procedures were performed in a three-arm style, with an 8 mm trocar for the camera placed 3 cm above the umbilicus and two 8 mm trocars on the right and left sides of the camera port. In addition, a 5 mm trocar was placed in the left lower quadrant for an accessory port. The port placement was similar in all cases. We selected the Maryland bipolar for the right arm and the fenestrated or force bipolar for the left arm. A uterine manipulator (Atom Medical, Tokyo, Japan) was inserted into the uterus preoperatively in all patients with benign diseases. The procedure consists of six steps, similar to a total laparoscopic hysterectomy [11]. Step one: The peritoneum of the vesicouterine pouch was incised from the cervix to the round ligament. We dissected the fascia of the retroperitoneal space and identified the ureter before resecting the fallopian tubes and infundibulopelvic ligament. This approach was performed to avoid ureteral injury. At that time, the uterine artery was identified and was ligated occasionally to reduce bleeding. Step two: The fallopian tubes and infundibulopelvic ligaments were handled. For tubectomy, we resected the fallopian mesentery and resected the ovarian ligaments. In the case of adnexectomy due to an ovarian tumor (cyst), we resected the infundibulopelvic ligament. While handling the fallopian tubes and infundibulopelvic ligament, we resected the round ligament. Following this, we resected the posterior leaf of the broad ligament toward the uterosacral ligament to expose the parametrium. Step three: The parametrium was transected. Occasionally, the uterine vessels of the parametrium were sutured to reduce backflow bleeding from the uterus. Step four: The vaginal wall was cut. Step five: The uterus was extracted transvaginally. Step 6: Following this, the vaginal wall and pelvic peritoneum were sutured closed.

We evaluated the skin-to-skin operative time, blood loss, postoperative hospital stay, intraoperative and postoperative complication rates of grade III or higher according to the Clavien-Dindo classification, conversion to laparotomy rate, and blood transfusion rate. Postoperative complications were defined as complications that occurred within 90 days of surgery. Blood loss was estimated by aspiration of intraoperative bleeding. 

All analyses were performed using the IBM SPSS Statistics, version 28.0(IBM Corp., Armonk, NY). We used the t-test and Kruskal-Wallis test to analyze continuous variables and the Χ^2^ test for categorical variables. Statistical significance was set at p < 0.05.

## Results

A total of 872 patients underwent robot-assisted hysterectomy during the study period. Of these, 148 patients met the exclusion criteria. The remaining 724 patients were analyzed. We classified these patients into four groups based on uterine weight: less than 250 g (377 patients), 250-500 g (253 patients), 500-750 g (69 patients), and 750 g or more (25 patients).

Table 1 shows the patients’ characteristics. The median uterine weight for each group was 157.3 g (<250 g), 340.5 g (250-500 g), 594.4 g (500-750 g), and 874.0 g (>750 g). The median BMI for each group was 23.6 kg/m^2^ (<250 g), 23.8 kg/m^2^ (250-500 g), 25.4 kg/m^2^ (500-750 g), and 23.7 kg/m^2^ (>750 g), showing significant differences between the groups (p = 0.031). The proportion of women with a delivery history in each group was 82.5% (<250 g), 80.6% (250-500 g), 62.3% (500-750 g), and 64.0% (>750 g), which differed significantly between the groups. Patients with a smaller uterus had more consistent histories of previous delivery. The proportion of women with a history of abdominal surgery in each group was 30.2% (<250 g), 30.0% (250-500 g), 34.7% (500-750 g), and 24.0% (>750 g), with no significant differences between the groups.

**Table 1:** Patient background

Background	Uterine weight				p-value
	<250 g (n = 377)	250-500 g (n = 253)	500-750 g (n = 69)	>750 g (n = 25)	
Age (years), median (range)	47.1 (26-75)	46.4 (33-76)	46 (32-55)	46.8 (40-54)	0.946
BMI (kg/m^2^), median (range)	23.6 (16.2-42.7)	23.8 (15.4-39.3)	25.4 (16.5-37.7)	23.7 (16.7-34.8)	0.031*
Parity, n(%)	311 (82.5)	204 (80.6)	43 (62.3)	16 (64)	<0.01*
History of abdominal surgery, n(%)	114 (30.2)	76 (30.0)	24 (34.7)	6 (24.0)	0.77
Uterine weight (g), median (range)	157.3 (30-245)	340.5 (250-495)	594.4 (500-743.5)	874 (750-1090)	<0.01*

The surgical outcomes of the patients are shown in Table 2. The median operative time in each group was 74.0 min (<250 g), 86.6 min (250-500 g), 107.1 min (500-750 g), and 127.2 min (>750 g), showing significant differences between the groups. Operative time significantly increased with increased uterine weight (p < 0.01). The median blood loss in each group was 26.3 mL (<250 g), 55.4 mL (250-500 g), 81.5 mL (500-750 g), and 136.0 mL (>750 g), significantly differing between the groups. Similar to operative time, blood loss significantly increased with increased uterine weight (p < 0.01). Postoperative hospital stay was not significantly different between the groups (p = 0.448). The complication rate in each group was 1.6% (<250 g), 1.6% (250-500 g), 0% (500-750 g), and 0% (>750 g), with no significant difference between the groups (p = 0.679). The intraoperative complication rate for each group was 0.3% (<250 g), 0.4% (250-500 g), 0% (500-750 g), and 0% (>750 g) (p = 0.942), and the postoperative complication rate was 1.3% (<250 g), 1.2% (250-500 g), 0% (500-750 g), and 0% (>750 g) (p = 0.745). Details of complications are shown in Table 3. All intraoperative complications are intestinal injuries, whereas almost all postoperative complications are vaginal troubles. The readmission rate for each group was 0.3% (<250 g), 0.8% (250-500 g), 0% (500-750 g), and 0% (>750 g), with no significant difference (p = 0.691). No cases of conversion to laparotomy or blood transfusion were observed.

**Table 2:** Surgical outcomes

Outcomes	Uterine weight				p-value
	<250 g (n = 377)	250-500 g (n = 253)	500-750 g (n = 69)	>750 g (n = 25)	
Operative time (min), median (range)	74 (23.0-157.0)	86.6 (43.0-160.0)	107.1 (60.0-172.0)	127.2 (76.0-190.0)	<0.01*
Estimated blood loss (mL), median (range)	26.3 (0-680.0)	55.4 (0-1000.0)	81.5 (0-530.0)	136 (0-550.0)	<0.01*
Length of stay, median (range)	4.2 (3-31)	4.3 (3-29)	4.4 (4-18)	4.5 (3-9)	0.448
Complication, n(%)	6 (1.6)	5 (2.0)	0	0	0.679
Intraoperative, n(%)	1 (0.3)	1 (0.4)	0	0	0.942
Postoperative, n(%)	5 (1.3)	4 (1.6)	0	0	0.745
Readmission, n(%)	1 (0.3)	2 (0.8)	0	0	0.691
Conversion to laparoscopic surgery, n(%)	0	1 (0.4)	0	2 (8.0)	<0.01*
Conversion to laparotomy, n(%)	0	0	0	0	-
Transfusion, n(%)	0	0	0	0	-

**Table 3:** Complications

Complication	Uterine weight	Age	BMI	History of abdominal surgery	Complication Detail	Treatment
Intraoperative	180	73	23.8	None	Small intestine injury	Repair during surgery
	260	47	20.6	Appendectomy	Sigmoid colon injury	Reoperation
Postoperative	120	61	29.3	None	Vaginal bleeding	Hemostasis in outpatient
	140	39	25.5	Laparoscopic adnexectomy	Vaginal bleeding	Hemostasis in outpatient
	190	50	28.8	C-section	Surgical site infection	Hemostasis in outpatient
	200	45	23.7	C-section	Vaginal stump abscess	Readmission transvaginal drainage
	230	41	18.5	None	Vaginal bleeding	Hemostasis in outpatient
	260	48	26.4	C-section laparoscopic myomectomy	Port-site bleeding	Hemostasis in outpatient
	365	49	18.6	None	Vaginal stump dehiscence	Readmission repair
	420	45	17.7	None	Vaginal stump hematoma	Readmission transvaginal drainage

## Discussion

Our retrospective cohort study showed a significant increase in operative time and blood loss for robot-assisted hysterectomy with greater uterine weight, as well as similar complication rates and postoperative hospital stay regardless of uterine weight. These results are consistent with those of previous studies. 

Previous studies have compared operative times for robot-assisted hysterectomy based on uterine weight. Akazawa et al. [5] classified 527 patients into five groups according to their uterine weight and reported operative times of 123 min (<250 g), 130 min (250-500 g), 144 min (500-750 g), 180 min (750-1000 g), and 170 min (>1000 g). In a study by Nozaki et al. [8] in which a similar classification to that in our study was used, operating times of 88, 87, 104, and 127 min were reported for the <250 g, 250-500 g, 500-750 g, and ≥750 g groups, respectively, indicating that surgical time increases with greater uterine weight. There are many possible reasons for this increase. First, a large uterus makes it difficult to maintain the field of view during surgery and limits instrument manipulation [9]. Although not investigated in this study, it is also thought that a large uterus requires a longer time for extraction [6,7]. Perutelli et al. [12] measured morcellation time and reported that the time required for morcellation increased significantly with increased uterine weight. Regarding the amount of blood loss, several previous studies [5-7] have shown that the amount of blood loss increases with greater uterine weight. However, Carbonnel et al. [7] found no significant difference in hemoglobin levels with increased blood loss, suggesting a subtle difference. In addition, no cases of transfusion were observed in this study, suggesting that this difference was not clinically important. 

In our study, complications of Clavien-Dindo classification grade III or higher affected 1.4% (10/724) of the women, with two intraoperative and eight postoperative complications (Table 3). Similar to previous studies [2,5,7], the complication rate did not increase with increased uterine weight in our study. Intraoperative complications included gastrointestinal injuries in both cases, wherein one was a small bowel injury that was repaired intraoperatively, and the other was a sigmoid colon injury that was detected postoperatively and repaired by a re-operation. In our study, the correlation between uterine weight and gastrointestinal injury was unclear; however, Elessawy et al. [13] reported that rectal injury may increase with increased uterine weight. Previous studies on total hysterectomy [3,5,8] have reported urinary tract injuries, such as ureteral and bladder injuries, as intraoperative complications; however, our study did not report any of these complications. Although the influence of severe endometriosis, a high-risk factor for urinary tract injury, cannot be ruled out, previous studies [3,5] have found no relationship between uterine weight and ureteral or bladder injury. The most common postoperative complication was vaginal bleeding (three cases), and other complications included hematoma at the vaginal stump, vaginal cuff dehiscence, vaginal cuff abscess, port site wound infection, and port site wound bleeding. Orady et al. [2] also reported one case of vaginal bleeding, but vaginal bleeding showed no relationship with uterine weight. Although vaginal cuff sutures and hemostatic manipulation are possible factors associated with vaginal bleeding and hematoma, both maneuvers are performed after uterine resection. Therefore, there appears to be no relationship between uterine weight and vaginal bleeding. The risk factors for vaginal cuff dehiscence are transvaginal suture when compared with laparoscopic surgery, robotic surgery, or sutures with normal absorbable thread when compared with barbed sutures [14], and no previous study has shown that increased uterine weight is a risk factor for vaginal cuff dehiscence. No association was observed between uterine weight and surgical site infection. In conventional laparoscopic surgery, complication rates have been reported to rise as uterine weight increases [15], suggesting that robotic surgery may be more useful in larger uteri [9,16].

Conversion to laparotomy is a serious complication of minimally invasive surgery. Some reported that increased uterine weight increased the frequency of conversion [2,5], while others reported that uterine weight did not affect the frequency of conversion [7]. The reasons for conversion include massive bleeding, severe adhesions, and hesitation about morcellation due to suspected malignancy [17]. In our study, there were no cases of conversion to laparotomy. Instead, we observed three cases of laparoscopic conversion. Two of these cases were caused by massive bleeding from the parametrium. The remaining case was due to mechanical trouble with the da Vinci surgical system. In a study by Akazawa et al. [5], the conversion rates were 0% (<250 g), 1.9% (250-500 g), 5.1% (500-750 g), 11% (750-1000 g), and 11% (>1000 g), with a significantly increased risk of conversion to laparotomy for uterine weights of 750 g or more (p < 0.001). 

High-volume surgeons or hospitals have lower complication rates for hysterectomies, whereas low-volume surgeons or hospitals have higher complication rates [18]. In addition, conversion to laparotomy is less common in robot-assisted hysterectomies and laparoscopic hysterectomies performed by high-volume surgeons [19]. The exact boundary between high and low volumes varies from one study to another. Baba et al. [20] classified the number of robotic-assisted hysterectomies per year as low (nine or less), intermediate (10-25), and high (26 or more). Some studies have defined surgeon volume as 30 or more surgeries per year [21], but a systematic review by Mowat et al. [22] stated that a surgeon is a high-volume surgeon if they perform at least one surgery per month, and several studies on robotic surgery [23] have adopted this classification. Using this classification, six of the seven surgeons in our study were high-volume surgeons, and one was a low-volume surgeon. Although we did not intentionally allocate low-risk patients to low-volume surgeons, the 10 patients in our study who experienced complications of Clavien-Dindo grade III or higher and the two patients who underwent laparoscopic conversion for massive bleeding were managed by high-volume surgeons. Therefore, surgeon volume bias did not influence the rates of complications or conversion in our study. Moreover, our high-volume center had the “field effect,” which is the added value of high-volume hospitals that allows low-volume surgeons to achieve similar surgical outcomes compared with high-volume surgeons [24]. 

Our study has several strengths. First, as previously mentioned, this study had the largest sample among studies that have examined the effect of uterine weight on robot-assisted hysterectomy. Second, although this was a single-center study, it utilized the surgical outcome data of seven surgeons, implying that there was relatively little bias among the surgeons. However, the study also had limitations. First, its design was single-center and retrospective. Second, the uterine weight in most women was less than 1 kg, and we excluded cases of severe adhesion due to endometriosis, which may have led to a selection bias. In addition, the study was a univariate analysis, but a multivariate analysis is desirable to exclude the effect of confounding factors, such as BMI and history of delivery, which were significantly different between the groups.

## Conclusions

In this retrospective study, operative time, blood loss, and laparoscopic conversion from robot-assisted hysterectomy increased with greater uterine weight, but the complication rate and the length of postoperative hospital stay were similar. Further studies, including prospective randomized controlled trials, are required to determine the optimal modality (laparoscopic or robotic surgery) for each uterine weight.
